# Chemical Composition and Biological Activities of the Leaf Essential Oils of *Curcuma longa*, *Curcuma aromatica* and *Curcuma angustifolia*

**DOI:** 10.3390/antibiotics11111547

**Published:** 2022-11-03

**Authors:** Jawaher J. Albaqami, Hamida Hamdi, Arunaksharan Narayanankutty, Naduvilthara U. Visakh, Anju Sasidharan, Aswathi Moothakoottil Kuttithodi, Ademola C. Famurewa, Berin Pathrose

**Affiliations:** 1 Department of Biology, College of Science, Taif University, Taif 21944, Saudi Arabia; 2 Zoology Department, Faculty of Science, Cairo University, Giza 12613, Egypt; 3 Division of Cell and Molecular Biology, PG & Research Department of Zoology, St. Joseph’s College (Autonomous), Calicut 673008, India; 4 Department of Agricultural Entomology, College of Agriculture, Kerala Agricultural University, Thrissur 680656, India; 5 Department of Medical Biochemistry, Faculty of Basic Medical Sciences, College of Medicine, Alex-Ekwueme Federal University Ndufu-Alike Ikwo, Abakaliki 482131, Nigeria

**Keywords:** *Curcuma longa*, *Curcuma aromatica*, *Curcuma angustifolia*, essential oil, agricultural waste, antibacterial activity, cytotoxicity

## Abstract

*Curcuma* species are widely used as a food additive and also in various medicinal purposes. The plant is a rich source of essential oil and is predominantly extracted from the rhizomes. On the other hand, the leaves of the plants are usually considered as an agrowaste. The valorization of these *Curcuma* leaf wastes into essential oils is becoming accepted globally. In the present study, we aim to extract essential oils from the leaves of *Curcuma longa* (LEO), *C. aromatica* (REO), and *C. anguistifolia* (NEO). The chemical composition of these essential oils was analyzed by GC-MS. Free radical scavenging properties were evaluated against the radical sources, including DPPH, ABTS, and hydrogen peroxide. The antibacterial activity was assessed by the disc diffusion method and Minimum inhibitory concentration analysis against Gram positive (*Staphylococcus aureus*) and Gram negative (*Escherichia coli*, *Pseudomonas aeruginosa* and *Salmonella enterica*) bacteria. Results identified the compounds α-phellandrene, 2-carene, and eucalyptol as predominant in LEO. The REO was predominated by camphor, 2-bornanone, and curdione. The main components detected in NEO were eucalyptol, curzerenone, α-lemenone, longiverbenone, and α-curcumene. Antioxidant properties were higher in the LEO with IC_50_ values of 8.62 ± 0.18, 9.21 ± 0.29, and 4.35 ± 0.16 μg/mL, against DPPH, ABTS, and hydrogen peroxide radicals. The cytotoxic activity was also evident against breast cancer cell lines MCF-7 and MDA-MB-231 cells; the LEO was found to be the most active against these two cell lines (IC_50_ values of 40.74 ± 2.19 and 45.17 ± 2.36 μg/mL). Likewise, the results indicated a higher antibacterial activity for *Curcuma longa* essential oil with respective IC_50_ values (20.6 ± 0.3, 22.2 ± 0.3, 20.4 ± 0.2, and 17.6 ± 0.2 mm). Hence, the present study confirms the possible utility of leaf agrowastes of different *Curcuma* spp. as a possible source of essential oils with pharmacological potential.

## 1. Introduction

Herbal medicines are important tools in the management of health from the ancient days; traditional medicines and folk medicinal systems utilized these plants and plant products [1]. Among the various plants, the predominant ones include the spices that are the part of the daily diet [2]. Several such spices are widely utilized in Ayurvedic and Chinese traditional medicines as dietary agents in the management of infectious and chronic illness [3,4]. Bioactive compounds and extracts from various aromatic plants are known for their biological activities, including antimicrobial properties [5,6]. Members of Zingiberaceae, Apiaceae, Lamiaceae, and Myrtaceae are well-known spices with potential health benefits. The predominant spices include turmeric, ginger, clove, cinnamon, and ajwain; these spices are used in flood and medicines. Among these, the predominant ones belong to the Zingiberaceae; the different spices, including *Curcuma* and *Zingiber* genus, are widely studied ones.

*Curcuma* spp. are well described for their phytochemistry, pharmacological, and biological properties. Different species including *C. longa*, *C. aromatica*, *C. aeruginosa*, *C. amada*, and *C. xanthorrhiza* are utilized in pharmaceutics, cosmetics, and other industries. The cosmetic uses of the *Curcuma* spp. are well explored; the extract of the *C. longa* extract indicated the potential of improving skin color [7]. Likewise, *Curcuma mangga* extracts are shown to protect against the oxidative stress-associated ageing in fibroblast cells [8]. Likewise, the extracts of *C. aromatica* and *C. comosa* prevented the ultraviolet-induced oxidative damage and matrix mellatoproteinase expression in skin cells [9]. The pharmacological properties of the different species of *Curcuma* spp. are also evaluated in different disease models. In Parkinson’s disease, the *Curcuma longa* was found to be effective by preventing the apoptotic death of dopamine producing cells in substantia nigra [10]. The oral consumption of curcumin has been found to improve the cognitive aspects of Alzheimer’s patients [11,12]. Apart from these, the *Curcuma* spp. is also effective against metabolic disease including non-alcoholic fatty liver disease. The turmeric has been effective in regulating the hepatic hyperlipidemia and reducing NAFLD complications [13]. Clinical studies also confirmed the potential of *Curcuma* spp. and their isolated bioactive compounds [14,15].

The essential oils are other important compounds that are produced from the *Curcuma* rhizomes and leaves. The predominant compounds present in the rhizome essential oils of different species of *Curcuma* include Curzerenone and 14-hydroxy-δ-cadinene [16]. Further, the essential oils derived from the *Curcuma* and *Zingiber* are widely utilized for pest repellence and medicinal purpose. The essential oil of *C. longa* and *C. aromatica* are found to be biologically active in preventing the growth of bacterial communities and cancer cells [17]. In addition, the essential oils of *C. longa* was found to inhibit the mutagenesis and subsequently prevent the carcinogenesis in murine models [18,19]. Apart from these, the *C. aromatica* has been demonstrated to exert antioxidant effects by scavenging reactive radicals [20].

Primarily, the rhizomes of these plants are utilized in medicine and food; however, the leaves of the plants are usually considered and left alone as an agrowaste. Agro-wastes are the emerging concerns in the agriculture sector, which often increase the concern of pollution and other issues. The agricultural waste products include the residues of grains and crops, litter from leaf and plant parts, and the excretory material from livestock or poultry [21,22]. The decaying and burning of these wastes will cause serious pollution issues in water, soil, and air [23]. Hence, the management of these agro-wastes is emerging as an important concern. Recent developments in the area have indicated that the conversion of these products to value-added components makes an economically beneficial and environmentally friendly method for waste management. Among the various value-added products, the essential oils are predominant ones that are mainly isolated from the agrowastes [24,25]. Hence, the present study aims to analyze the chemical composition and pharmacological activities of essential oils derived from three species of *Curcuma* viz., *C. longa*, *C. aromatica,* and *C. augustifolia.* It is expected that, by virtue of the bioactive compounds present, these essential oils may control the population of microbial communities and cancer cell survival.

## 2. Results

### 2.1. Determination of the Yield and Chemical Composition of Leaf Essential Oils by GC-MS

As shown in the Table 1, the yield of leaf essential oils of different *Curcuma* spp. varied much from the others. The highest yield was noticed in the *Curcuma longa* (1.62 ± 0.34%). However, the lowest level of yield was noticed in the *C. agnustifolia* (0.37 ± 0.02%).

**Figure 1:**
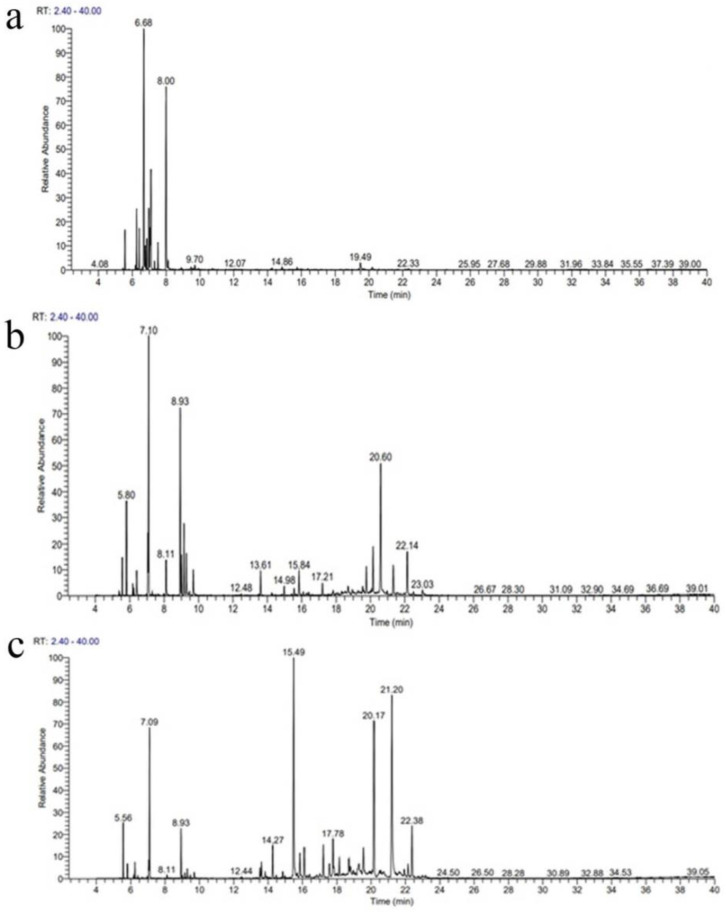
The chromatograms of GC-MS analysis of *C. longa* (**a**), *C. aromatica* (**b**), and *C. angustifolia* (**c**) leaf essential oil.

The gas chromatography- mass spectroscopy analysis revealed the presence of various stress volatiles in the leaf essential oils of different *Curcuma* spp. The chromatograms of all tested essential oils are shown in Figure 1. The chemical components and percentage composition of *Curcuma longa* (Supplementary Table S1), *C. aromatica* (Supplementary Table S2), and *C. angustifolia* (Supplementary Table S3) have been listed.

The composition of essential oils is presented in Table 2 and Tables S1–S3 from the Supplementary Material. The predominant compounds in LEO were α-phellandrene (31.27%), 2-carene (21.73%), eucalyptol (13.54%), and o-cymene (5.45%) (Supplementary Table S1). In REO, the predominant compounds were camphor (19.82%), 2-bornanone (12.25%), and curdione (15.31%) (Supplementary Table S2). On the contrary, in NEO, eucalyptol (11.58%), curzerenone (25.32%), α-lemenone (13.59%), longiverbenone (9.37%), boldenone (5.04%), and α-curcumene (5.12%) were the major compounds (Supplementary Table S3).

### 2.2. Antioxidant Activities of Leaf Essential Oils of Different Curcuma *spp.*

The antioxidant activities of the different leaf essential oils of LEO, REO, and NEO were estimated in terms of DPPH, ABTS, and hydrogen peroxide radical scavenging activities (Table 3). The LEO was found to be the most active among the tested essential oils in all the antioxidant assays. The respective IC_50_ values of LEO against the three radicals were estimated to be 8.62 ± 0.18, 9.21 ± 0.29, and 4.35 ± 0.16 μg/mL. On contrary, the REO and NEO had significantly higher IC_50_ values for the three radical scavenging assays in comparison with LEO (*p* < 0.05). The *Curcuma* essential oils demonstrated higher hydrogen peroxide scavenging abilities (*p* < 0.05). The LEO had similar DPHH and ABTS radical scavenging potential as that of ascorbic acid. The detailed statistical analysis of the antioxidant potential is shown in Supplementary Table S4.

Statistical analysis of the data is given in Supplementary Table S4.

### 2.3. Cytotoxic Activity of Leaf Essential Oil of Different Curcuma *spp.*

**Figure 2:**
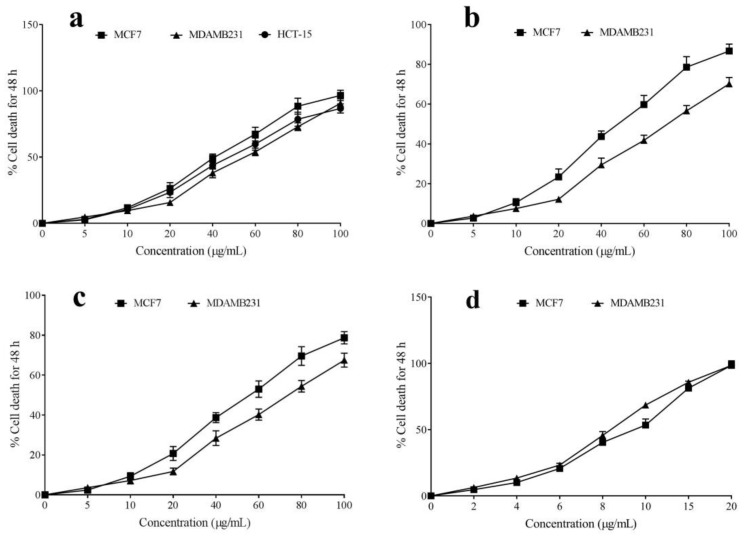
Cytotoxic activity of leaf essential oils extracted from *Curcuma longa* (**a**), *Curcuma aromatica*, (**b**) *Curcuma angustifolia* (**c**), and cyclophosphamide (**d**) against breast cancer cell lines—MCF7 (ER positive) and MDA-MB-231 (triple negative).

The anti-neoplastic properties of the leaf essential oils of different *Curcuma* spp. (LEO, REO, and NEO) were evaluated on human breast cancer lines. The result indicated strong cytotoxic properties in LEO, followed by REO and NEO; it is reflected in the IC_50_ values of the essential oils, with LEO having the lowest IC_50_ value and NEO being the highest (Table 4). In addition, the cytotoxicity in these cells was dose-dependent (Figure 2). Comparing among the two breast cancer cell lines used, all the tested essential oils had higher toxicity in MCF-7 cells than MDA-MB-231.

The half-maximal inhibition concentration of the leaf essential oils are listed in Table 4. The *C. longa* demonstrated an IC_50_ value of 40.74 ± 2.19 and 45.17 ± 2.36 μg/mL against MCF-7 and MDA-MB-231. The lowest cytotoxicity was exhibited by *C. angustifolia* with IC_50_ values of 64.17 ± 1.95 and 70.31 ± 1.59 μg/mL, respectively, against MCF-7 and MDA-MB-231 cells. The cyclophosphamide was more toxic to these cells with respective IC_50_ values of 9.46 ± 0.20 and 8.52 ± 0.22 μg/mL. The detailed statistical analysis among essential oils is shown in Supplementary Table S5.

Statistical analysis of the data is given in Supplementary Table S5.

### 2.4. Antibacterial Activity of Leaf Essential Oil of Different Curcuma *spp.*

The antibacterial activity was estimated by the disc-diffusion method. *Curcuma longa* essential oil was the most active against all the selected bacterial strains (Table 5). The inhibition zone of LEO was found to be high against *Staphylococcus aureus* and *Pseudomonas aeruginosa* (*p* < 0.01). The REO was effective against *E. coli*, being statistically significant over NEO, but was, however, lower than the LEO. Among the three tested essential oils, the weakest one was from *C. angustifolia*. The standard gentamicin is also found to be highly effective against these bacterial strains with respective inhibition zones of 22.4 ± 0.3, 19.7 ± 0.1, 22.5 ± 0.3, and 19.1 ± 0.5 mm against the different bacteria (Table 5). The complete statistical analysis is listed in Supplementary Table S6.

Statistical analysis of the data is given in Supplementary Table S6; *Curcuma longa* (LEO), *C. aromatica* (REO), and *C. angustifolia* (NEO).

The antibacterial activity was also estimated in terms of the MIC values; among the three essential oils tested, the LEO was found to have significant antibacterial activity. In addition, the LEO and REO were equally effective against *Pseudomonas aeruginosa* with similar MIC values (Table 6). On comparing with the essential oils, the gentamicin treatment was more effective in terms of the MIC values; more statistical operations and details are listed in the Supplementary Table S7.

Statistical analysis of the data is given in Supplementary Table S7; *Curcuma longa* (LEO), *C. aromatica* (REO), and *C. angustifolia* (NEO).

## 3. Discussion

Spices are important dietary components with potential biological and pharmacological activities [26]. Spices are highly utilized in food industries and therefore it is an important source of biologically active molecules that are referred to as nutraceuticals [27]. Among the various spices used, the Turmeric (*Curcuma longa*) is considered to be the most accepted one [28,29]. Apart from the *C. longa*, there are several other species that exist in the genus. In the present study, we evaluated the chemical composition of the leaf essential oils of different *Curcuma* spp., which is considered to be the important agrowaste. Apart from these, the antibacterial and cytotoxic activities were also evaluated.

Our results indicated a yield between 0.37 to 1.62% for the different *Curcuma* spp. essential oils. However, previous studies by Kutti Gounderand and Lingamallu [30] and Hong, et al. [31] indicated a yield of 3.05 to 4.45% from rhizomes. However, considering that the present study used leaves as the source of essential oil, a yield of 1.62% may not be considered low.

The three essential oil contains entirely different chemical composition; In LEO, α-phellandrene, 2-carene, and eucalyptol predominated the chemical contents; whereas, the REO was predominated by camphor, 2-bornanone, and curdione. The main components detected in NEO were eucalyptol, curzerenone, α-lemenone, longiverbenone, and α-curcumene. Previous studies by Jena, Ray, Banerjee, Sahoo, Nasim, Sahoo, Kar, Patnaik, Panda, and Nayak [16] indicated the presence of curzerenone (33.2%), 14-hydroxy-δ-cadinene (18.6%) and γ-eudesmol acetate (7.3%) in the *C. angustifolia* leaf essential oil. Chemical analysis of *C. longa* leaf essential oil in the studies of Sindhu, et al. [32] and Sharma, et al. [33] indicated the presence of phellandrene, eucalyptol, p-cymene, terpinolene, and β-pinene. According to the previous reports, in *C. aromatica* leaf essentila oil, the main components are eucalyptol (20.0%), camphor (18.0%) germacrone (11.8%), camphene (9.4%), limonene (8.6%), and isoborneol (6.4%) [20]. Hence, previous reports are also in line with our study; however, the percentage composition shows a significant variation.

Besides the chemical constituent analysis, the results also indicated strong free radical quenching potential. The previous reports by Avanço, et al. [34] and Jena, Ray, Banerjee, Sahoo, Nasim, Sahoo, Kar, Patnaik, Panda, and Nayak [16] indicated the antioxidant properties of the rhizome essential oils of different *Curcuma* spp. Likewise, the radical quenching properties are also attributed to the *Curcuma* leaf essential oils [17,35]. Furthermore, the bioactive compounds including eucalyptol, α-lemenone, α-phellandrene, 2-carene and α-curcumene are also reported to act as chain breaking antioxidants [36,37]. Since the role of antioxidants in alleviating chronic diseases and preventing infectious disease are evident [38,39], the leaf essential oils from different *Curcuma* spp. may also have significant health promoting effects.

The present results also indicated strong cytotoxic properties against breast cancer cells. Cytotoxic activity of the essential oil of *C. aromatica* has also been evident in multiple cancer cells by stimulating apoptotic cell death [40,41]. Previous studies have also been reported that the essential oils of *Curcuma* rhizome and leaves induce apoptotic cell death in lung and liver cancer cells [42,43]. The cytotoxicity is attributed to the specific compounds such as α-phellandrene [44,45], camphor [46], curdione [47,48], eucalyptol [49,50], terpinolene [51], and α-pinene [52,53], which are already known to induce apoptosis and signaling interruption in cancer cell. The cytotoxic effect was mediated through cell cycle inhibition at the G_2_/S checkpoint [54].

Results also indicated strong antimicrobial properties to the leaf essential oils of different *Curcuma* spp. against bacterial strains such as *E. coli*, *S. aureus*, and *S. enterica*. These pathogenic microbes are known to cause various health issues in humans and animals [55,56]. The antibacterial activity was also attributed to the different *Curcuma* essential oils; previous studies have indicated the antibacterial properties of *C. longa* [57,58], *C. aromatica* [59,60], *C. angustifolia* [61]. The antibacterial activity of *C. longa* rhizome essential oil is also evident against *Bacillus subtilis*, *Staphylococcus aureus*, *Salmonella typhimurium*, and *Escherichia coli* [34,57]. Likewise, the rhizome essential oil of *C. aromatica* is also found to be effective against various microorganisms [60]. In addition, the essential oils were also capable of inhibiting the biofilm forming properties of bacteria, including *Streptococcus mutans* [62]. A recent study by Septama, et al. [63] has also indicated the antibacterial and anti-biofilm formation activities of the *C. xanthorrhiza*. To support this information, there are bioactive constituents, such as α-phellandrene [64,65], camphor [66,67,68], eucalyptol [69], terpinolene [70], and α-pinene [52,71].

Hence, the present study indicated significant variation in the chemical composition of the leaf essential oils of different *Curcuma* spp. Further, these essential oils displayed significant radical quenching potential against DPPH, ABTS, and peroxide radicals. The essential oils, especially LEO, exhibited strong antibacterial properties against Gram positive and Gram-negative strains. The cytotoxic activities of the different *Curcuma* essential oils were also identified against breast cancer cells.

## 4. Materials and Methods

### 4.1. Materials and Chemicals

The chemicals used for the analysis were of reagent grade and purchased from Sigma Aldrich (St. Louis, MO, USA). The chemicals were DPPH, ABTS, hydrocarbon mixture (C8–C30 n-alkanes), ethanol, and hydrogen peroxide. Cell culture reagents include Dulbecco’s Modified Eagle Media, sodium pyruvate, fetal bovine serum, non-essential amino acids, and MTT (Gibco, MA, USA). The microbial growth media included Lysogeny broth and Mueller–Hinton agar (Himedia, Mumbai, India).

The human breast cancer cell lines (MCF-7, and MDA-MB-231) were procured from National Centre for Cell Science, Pune. The bacterial strains were obtained from the Microbial Type Culture Collection and Gene Bank (MTCC), Chandigarh, India. The Gram-positive bacteria used was *Staphylococcus aureus* (MTCC740) and Gram-negative bacteria were *Escherichia coli* (MTCC1610), *Pseudomonas aeruginosa* (MTCC 741), and *Salmonella enterica* (MTCC1252) bacteria.

### 4.2. Collection of Curcuma Leaves and Extraction of Essential Oil

Leaves of different species of *Curcuma* spp. belonging to the Zingiberaceae family were collected from Kerala Agricultural University, Thrissur, India (10.85053° N, 76.27106° E) in February 2022. Hydro-distillation was conducted to extract the essential oils from these different species of *Curcuma* spp. with the help of the modified Clevenger-type apparatus for 5–6 h (100 °C). Briefly, 20 g of *Curcuma* leaves alone were taken in a 2000 mL flask along with deionized water. Initially, they were heated to boil as described in Table 1; the essential oil was then collected and dried with anhydrous Na_2_SO_4_ [72]. Finally, these essential oils were stored in dark amber-colored glass bottles at 4 °C inside the refrigerator until required for experiments. The essential oil yield was determined on a dry weight basis by using the formula yield (%, *v*/*w*).

$$Percentage\;Yied=\frac{Volume\;of\;dry\;essential\;oil}{Weight\;of\;shade\;dry\;leaves}\times 100$$

### 4.3. Chemical Component Analysis by GC-MS Analysis

The chemical composition of the essential oils were determined according to our previously published method [73]. The chromatographic equipment used in the analysis was TSQ 8000 Evo system from the Thermo scientific (Waltham, MA, USA). The analytical system was composed of an autosampler, which was a gas chromatographic column (TG-5MS) of dimensions 30 mm × 0.25 mm × 0.25 μm. The helium gas was used as a carrier with 1.0 mL per minute flow rate. The gas chromatographic oven was maintained at 50 °C with a gradual and steady increase to 120 °C (10 °C per minute) and finally changing the temperature to 270 °C (at a rate of 5 °C per minute). The chemical composition of each essential oil was derived by matching the MS spectra of NIST library. Each run was followed by a blank run without essential oil to omit the carry over contamination. We determined the retention index (RI) values by calibrating the instrument with a homologous series of alkanes (C_7_–C_30_ n-alkane mixture) using the same conditions. The calculated retention indices of identified chemical components were compared with library reference retention indices in NIST and Wiley libraries [74,75,76,77].

### 4.4. In Vitro Antioxidant Activity as Scavenging of DPPH, Hydrogen Peroxide and ABTS Radicals of the Essential Oils of Different Curcuma *spp.*

The in vitro antioxidant activities were estimated for the selected essential oils; the essential oils were initially diluted to appropriate concentrations (0–25 μg/mL) and used for the study.

#### 4.4.1. Anti-DPPH Radical Assay

The 2,2-diphenyl-1-picrylhydrazyl (DPPH) radical scavenging was estimated according to the methods of Baliyan, Mukherjee, Priyadarshini, Vibhuti, Gupta, Pandey, and Chang [26]. Briefly, the varying concentrations of the essential oils were mixed with DPPH (0.12 M) solution in methanol. The mixture was kept in dark at 30 °C for 20 min. The reduction in the optical densities in different essential oil doses was compared to the untreated control, and the percentage inhibition was calculated using the formula;

$$\%\;Inhibition=\frac{OD\;of\;Control-OD\;of\;Sample}{OD\;of\;Control}\times 100$$

#### 4.4.2. Curcuma Essential Oils and ABTS Radical Quenching Ability

The 2,2′-azino-di-(3-ethylbenzthiazoline sulfonic acid) (ABTS) radical scavenging was estimated according to the methods of Munteanu and Apetrei [78]. Initially, the ABTS radicals were generated by incubating 8 mM ABTS and 2.5 mM potassium persulfate for 12 h at 30 °C. These radicals were diluted 1:60 to a yield working ABTS solution; 1 mL of this solution was mixed with different doses of *Curcuma* essential oils and incubated at 30 °C for 10 min, and the change in absorbance was noted at 734 nm using the same formula given in Section 4.4.1

#### 4.4.3. Hydrogen Peroxide Neutralization Assay

The hydrogen peroxide scavenging was estimated according to the methods described by Al-Amiery, et al. [79]. The essential oil of varying concentration (0.1 mL) was mixed with 50 mM phosphate buffer of pH 7.4 ± 0.1, containing 2 mM hydrogen peroxide solution. The mixture was mixed and incubated in the dark for 10 min at 30 °C, and the change in absorbance was noted at 234 nm.

### 4.5. Cytotoxic Activity of the Leaf Essential Oils of Different Curcuma *spp.*

Two breast cancer cell lines- MCF7 (ATCC, HTB-22™ Estrogen receptor positive) and the MDA-MB-231 (ATCC, HTB-26™ triple negative breast cancer cell) were procured from National Centre for Cell Science (Pune, Maharashtra, India). These cells were maintained in complete Dulbecco’s Modified Eagle Media (Gibco, MA, USA) with sodium pyruvate, sodium carbonate, and non-essential amino acids. The cytotoxicity analysis was conducted according to the MTT assay, as mentioned previously [80]. The percentage of cell death was estimated by the formula;

$$\%\;Cell\;death=\frac{OD\;of\;Control-OD\;of\;Sample}{OD\;of\;Control}\times 100$$

OD means the optical density or absorbance.

### 4.6. Antibacterial Activity of Leaf Essential Oil of Different Curcuma *spp.* by Disc Diffusion Method

The bacterial strains were initially maintained in Lysogeny broth (Himedia, Mumbai, India); the bacteria were inoculated in an Mueller–Hinton agar (Himedia, Mumbai, India) plate of thickness 5 mm. The Whatman No.1 filter paper disc of 8 mm diameter was immersed with the different *Curcuma* essential oils (10 μL). These filter paper discs were placed in different parts of the plates at a distance of 50 mm diameter apart in a plate. These plates were incubated at 37 °C for 24 h and the zone of inhibition was estimated for each bacterial strain [81].

### 4.7. Minimum Inhibitory Concentration (MIC) of Essential Oil of Different Curcuma *spp.*

The MIC value was estimated by the previously described methods [82,83,84]. Briefly, the bacterial inoculum density was maintained to 5 × 10^5^ CFU/mL by the spectrophotometry. Further, the 50 μL of inoculum was placed in a 96-well plate and mixed with different concentrations of different *Curcuma* essential oils prepared in 0.1% agar. The media was then mixed with 10 μL of 2,3,5-triphenyltetrazolium chloride (TTC), a pink dye, which loses its color in the absence of microbial growth. The lowest concentration without pink color (confirmed with control group using spectrophotometer) was estimated to be the MIC value.

### 4.8. Statistical Analysis

The values of antioxidant activity, cytotoxicity, and antimicrobial assays were expressed as mean ± standard deviation of six individual analyses, which are carried out in triplicate. The IC_50_ values were calculated using the Probit analysis method in the GraphPad prism. The statistical analysis was carried out by the analysis of variance using GraphPad prism ver. 7.0 (La Jolla, CA, USA).

## 5. Conclusions

The results indicated that the leaf waste materials of the *Curcuma* spp. can be effectively converted to essential oils. The yield of the oil was also good, considering the source of extraction is a waste product. The predominant compounds in *C. longa* essential oil were α-phellandrene and 2-carene; whereas camphor and curdione predominated in the *C. aromatica* essential oil and curzerenone and α-elemenone was high in the *Curcuma angustifolia* essential oil. The highest antioxidant potential, cytotoxicity and antibacterial activity were exhibited by the *C. longa* essential oils, followed by *C. aromatica*. Taking together, it is assumed that the leaf wastes of *Curcuma* spp., especially *C. longa*, can be converted to commercially useful essential oils with antioxidant, cytotoxic, and antibacterial properties.

## Figures and Tables

**Table 1:** Yield and extraction method of essential oils obtained from different species of *Curcuma* spp. through hydro-distillation.

Species	Extraction Time (Hour)	Fresh Weight (kg)	Yield% (*v*/*w*)	Color
*Curcuma longa*	5	1.8	1.62 ± 0.34	Light brown
*Curcuma aromatica*	5	1.1	0.51 ± 0.15	Light brown
*Curcuma angustifolia*	5	1.0	0.37 ± 0.02	Light brown

**Table 2:** The major compounds detected in the essential oil extracted from the leaves of different *Curcuma* spp. by GC-MS analysis.

*Curcuma* spp. Essential Oil	RT ^a^	Component	RI ^b^	RI ^c^	%RA ^d^
*C. longa*	5.56	β-Pinene	981	980	4.76
	6.68	α-Phellandrene	1006	1004	31.27
	6.76	o-Cymene	1029	1030	5.45
	7.10	Eucalyptol	1050	1052	13.54
	8.00	2-Carene	1148	1168	21.73
*C. aromatica*	5.80	Camphene	956	955	4.80
	7.09	Camphor	1135	1134	19.82
	8.93	2-Bornanone	1144	1145	12.25
	9.15	Isoborneol	1154	1153	4.56
	20.59	Curdione	1679	1680	15.31
	22.14	1-heptatriacotanol	1683	1688	4.70
*C. angustifolia*	7.09	Eucalyptol	1027	1029	11.58
	13.62	α-Curcumene	1470	1462	5.12
	15.49	Curzerenone	1499	1488	25.32
	18.15	Boldenone	1570	1574	6.45
	20.17	α-Elemenone	1670	1670	13.59
	21.20	Longiverbenone	1676	1678	9.37

**^a^** Retention time; **^b^** Retention index (library); **^c^** Retention index (calculated); **^d^** Relative area.

**Table 3:** Antioxidant activities of the *Curcuma* spp. leaf essential oils—expressed as IC_50_ (μg/mL).

	DPPH Radical Scavenging	ABTS Radical Scavenging	H_2_O_2_ Radical Scavenging
*C. longa* (LEO)	8.62 ± 0.18	9.21 ± 0.29	4.35 ± 0.16
*C. aromatica* (REO)	15.23 ± 0.35	13.28 ± 0.51	8.38 ± 0.24
*C. angustifolia* (NEO)	16.08 ± 0.22	12.81 ± 0.43	8.08 ± 0.31
Ascorbic acid	9.72 ± 0.15	10.97 ± 0.36	15.55 ± 0.29

**Table 4:** Cytotoxic activity of the *Curcuma* spp. leaf essential oils expressed as IC_50_ (μg/mL).

	MCF-7	MDA-MB-231
*C. longa* (LEO)	40.74 ± 2.19	45.17 ± 2.36
*C. aromatica* (REO)	55.75 ± 1.39	67.11 ± 3.07
*C. angustifolia* (NEO)	64.17 ± 1.95	70.31 ± 1.59
Cyclophosphamide	9.46 ± 0.20	8.52 ± 0.22

**Table 5:** Antibacterial activity of *Curcuma* spp. leaves essential oils evaluated by disc diffusion method.

Bacteria	Zone of Inhibition (mm)			
	LEO	REO	NEO	Gentamicin
*Escherichia coli*	20.6 ± 0.3	19.1 ± 0.2	17.5 ± 0.2	22.4 ± 0.3
*Pseudomonas aeruginosa*	22.2 ± 0.3	18.0 ± 0.3	16.8 ± 0.2	19.7 ± 0.1
*Staphylococcus aureus*	20.4 ± 0.2	16.3 ± 0.3	16.1 ± 0.2	22.5 ± 0.3
*Salmonella enterica*	17.6 ± 0.2	16.1 ± 0.1	15.5 ± 0.2	19.1 ± 0.5

**Table 6:** Antibacterial activity of *Curcuma* spp. leaves essential oils evaluated as minimum inhibitory concentrations (μg/mL) of leaf essential oils of different *Curcuma* spp.

Bacteria	MIC Concentration (mg/mL)			
	LEO	REO	NEO	Gentamicin
*Escherichia coli*	0.625 ± 0.02	1.000 ± 0.02	1.000 ± 0.01	0.0312 ± 0.00
*Pseudomonas aeruginosa*	0.625 ± 0.03	0.625 ± 0.02	0.750 ± 0.03	0.0312 ± 0.00
*Staphylococcus aureus*	0.500 ± 0.01	0.750 ± 0.04	1.000 ± 0.02	0.0625 ± 0.01
*Salmonella enterica*	0.625 ± 0.02	1.250 ± 0.03	1.250 ± 0.03*	0.0312 ± 0.00

## Data Availability

Data may be made available on valid request.

## Supplementary Materials

The following supporting information can be downloaded at: https://www.mdpi.com/article/10.3390/antibiotics11111547/s1, Supplementary Table S1. The major compounds detected in the essential oil extracted from the leaves of *C. longa* (LEO) species by GC-MS analysis; Supplementary Table S2. The major compounds detected in the essential oil extracted from the leaves of *C. aromatica* (REO) species by GC-MS analysis; Supplementary Table S3. The major compounds detected in the essential oil extracted from the leaves of *C. angustifolia* (NEO) species by GC-MS analysis; Supplementary Table S4. Statistical comparison of the antioxidant activities among different essential oils; Supplementary Table S5. Statistical comparison of the cytotoxic activities among different essential oils and the standard drug cyclophosphamide; Supplementary Table S6. Statistical comparison of the antibacterial activities among different essential oils and gentamicin; Supplementary Table S7. Statistical comparison of the antibacterial activities in terms of minimum inhibitory concentration among different essential oils and gentamicin
