# Supplementary material for: Chemical Composition and Biological Activities of the Leaf Essential Oils of Curcuma longa, Curcuma aromatica and Curcuma angustifolia
Source: Antibiotics (Basel). 2022 Nov 3;11(11):1547. doi: 10.3390/antibiotics11111547 (PMC9686912; doi:10.3390/antibiotics11111547)
Supplement: Supplementary file 1 [file antibiotics-11-01547-s001.zip › antibiotics-1988610-supplementary.pdf]

Supplementary Table S1. The major compounds detected in the essential oil extracted from the leaves of *C. longa* (LEO) species by GC-MS analysis

Peak no.	RT[a]	Component	RI[b]	RI[c]	%RA[d]
1	5.25	α-pinene	927	928	3.07
2	5.56	β-pinene	981	980	4.76
3	6.41	3-carene	1006	1004	2.10
4	6.68	α-phellandrene	1011	1010	31.27
5	6.76	o-cymene	1029	1030	5.45
6	6.85	α-myrcene	1042	1042	3.41
7	6.98	α-ocimene	1047	1048	2.69
8	7.10	Eucalyptol	1050	1052	13.54
9	7.51	cis-terpinene	1063	1063	2.50
10	8.00	2-carene	1148	1168	21.73
11	8.12	1,6-Octadien-3-ol, 3,7-dimethyl-	1184	1185	1.29
12	9.47	Terpinen-4-ol	1188	1197	0.49
13	9.58	Thymol	1266	1212	0.78
14	9.70	α- terpineol	1201	1215	0.53
15	12.07	Exo-2-Hydroxycineole	1228	1229	0.33
16	14.27	Caryophyllene	1407	1406	0.81
17	15.75	α-curcumene	1489	1475	0.88
18	16.04	Bisabolene	1524	1520	0.58
19	19.49	Tumerone	1634	1633	1.56
20	20.19	Curlone	1655	1654	0.58
21	22.34	Alloaromadendrene oxide	1685	1676	0.39
		Total identified compounds			98.16 %

[a] Retention time; [b] Retention index (library); [c] Retention index (calculated); [d] Relative area

Supplementary Table S2. The major compounds detected in the essential oil extracted from the leaves of *C. aromatica* (REO) species by GC-MS analysis

Peak no.	RT [a]	Component	RI [b]	RI [c]	%RA [d]
1	5.56	α-pinene	930	932	1.91
2	5.80	Camphene	956	955	4.80
3	6.40	α-myrcene	983	981	1.64
4	7.10	Camphor	1135	1134	19.82
5	8.11	Linalyl acetate	1139	1140	2.44
6	8.93	2-bornanone	1144	1145	12.25
7	9.01	Eucalyptol	1160	1151	2.47
8	9.15	Isoborneol	1154	1153	4.56
9	9.30	endo-Borneol	1162	1162	3.11
10	9.47	Terpinen-4-ol	1188	1169	0.89
11	9.69	α-terpineol	1179	1178	2.11
12	13.61	Humulene	1146	1146	1.94
13	14.98	cis- α-bisabolene	1498	1366	0.88
14	15.84	α-copaene	1378	1375	2.36
15	17.21	cis-elemene	1441	1440	1.11
16	17.83	α-guaiene	1459	1458	0.95
17	18.70	Spathulenol	1572	1570	1.14
18	19.76	Isoaromadendrene epoxide	1584	1580	2.43
19	20.15	Elemenone	1580	1582	4.46
20	20.59	Curdione	1679	1680	15.31
21	21.32	Neocurdione	1681	1682	3.07
22	22.14	1-heptatriacotanol	1683	1688	4.70
23	23.03	Aromadendrene oxide-(2)	1685	1691	0.76
		Total identified compounds			95.31 %

[a] Retention time; [b] Retention index (library); [c] Retention index (calculated); [d] Relative area

Supplementary Table S3. The major compounds detected in the essential oil extracted from the leaves of *C. angustifolia* (NEO) species by GC-MS analysis

Peak no.	RT [a]	Component	RI [b]	RI [c]	%RA [d]
1	5.56	α-pinene	930	933	2.31
2	5.80	camphene	956	952	1.57
3	7.09	Eucalyptol	1027	1029	11.58
4	8.93	2-Bornanone	1144	1146	2.33
5	9.70	α-terpineol	1179	1178	1.13
6	13.53	α-bourbonene	1411	1410	1.88
7	13.62	α-curcumene	1470	1462	5.12
8	14.27	Caryophyllene	1464	1465	1.17
9	14.85	cis- α-farnesene	1495	1496	0.87
10	15.49	Curzerenone	1499	1488	25.32
11	17.02	α-acorenol	1628	1552	0.89
12	17.21	β-elemene	1438	1555	1.40
13	17.78	Caryophyllene oxide	1582	1570	3.60
14	18.15	Boldenone	1570	1574	6.45
15	18.71	Spathulenol	1576	1577	1.41
16	20.17	α-elemenone	1670	1670	13.59
17	21.20	Longiverbenone	1676	1678	9.37
18	21.63	Alloaromadendrene oxide-(2)	1685	1687	3.99
		Total identified compounds			93.98 %

[a] Retention time; [b] Retention index (library); [c] Retention index (calculated); [d] Relative area

Supplementary Table S4. Statistical comparison of the antioxidant activities among different essential oils

Tukey's multiple comparisons test	Mean Diff.	95.00% CI of diff.	Significant?	Summary	Adjusted P Value
**DPPH radical scavenging**					
LEO vs. REO	-6.61	-7.307 to -5.913	Yes	****	<0.0001
LEO vs. NEO	-7.46	-8.157 to -6.763	Yes	****	<0.0001
LEO vs. Ascorbic acid	-1.1	-1.797 to -0.4034	Yes	**	0.0011
REO vs. NEO	-0.85	-1.547 to -0.1534	Yes	*	0.0128
REO vs. Ascorbic acid	5.51	4.813 to 6.207	Yes	****	<0.0001
NEO vs. Ascorbic acid	6.36	5.663 to 7.057	Yes	****	<0.0001
**ABTS radical scavenging**					
LEO vs. REO	-4.07	-4.767 to -3.373	Yes	****	<0.0001
LEO vs. NEO	-3.6	-4.297 to -2.903	Yes	****	<0.0001
LEO vs. Ascorbic acid	-1.76	-2.457 to -1.063	Yes	****	<0.0001
REO vs. NEO	0.47	-0.2266 to 1.167	No	ns	0.2709
REO vs. Ascorbic acid	2.31	1.613 to 3.007	Yes	****	<0.0001
NEO vs. Ascorbic acid	1.84	1.143 to 2.537	Yes	****	<0.0001
**H2O2 radical scavenging**					
LEO vs. REO	-4.03	-4.727 to -3.333	Yes	****	<0.0001
LEO vs. NEO	-3.73	-4.427 to -3.033	Yes	****	<0.0001
LEO vs. Ascorbic acid	-11.2	-11.9 to -10.5	Yes	****	<0.0001
REO vs. NEO	0.3	-0.3966 to 0.9966	No	ns	0.6401
REO vs. Ascorbic acid	-7.17	-7.867 to -6.473	Yes	****	<0.0001
NEO vs. Ascorbic acid	-7.47	-8.167 to -6.773	Yes	****	<0.0001

Supplementary Table S5. Statistical comparison of the cytotoxic activities among different essential oils and the standard drug cyclophosphamide

Tukey's multiple comparisons test	Mean Diff.	95.00% CI of diff.	Adjusted P Value
**MCF7**			
LEO vs. REO	-15.01	-17.91 to -12.11	<0.0001
LEO vs. NEO	-23.43	-26.33 to -20.53	<0.0001
LEO vs. Cyclophosphamide	31.28	28.38 to 34.18	<0.0001
REO vs. NEO	-8.42	-11.32 to -5.517	<0.0001
REO vs. Cyclophosphamide	46.29	43.39 to 49.19	<0.0001
NEO vs. Cyclophosphamide	54.71	51.81 to 57.61	<0.0001
**MDA-MB-231**			
LEO vs. REO	-21.94	-24.84 to -19.04	<0.0001
LEO vs. NEO	-25.14	-28.04 to -22.24	<0.0001
LEO vs. Cyclophosphamide	36.65	33.75 to 39.55	<0.0001
REO vs. NEO	-3.2	-6.103 to -0.2975	0.0258
REO vs. Cyclophosphamide	58.59	55.69 to 61.49	<0.0001
NEO vs. Cyclophosphamide	61.79	58.89 to 64.69	<0.0001

Supplementary Table S6. Statistical comparison of the antibacterial activities among different essential oils and gentamicin

Tukey's multiple comparisons test	Mean Diff.	95.00% CI of diff.	Adjusted P Value
Escherichia coli			
LEO vs. REO	1.5	1.105 to 1.895	<0.0001
LEO vs. NEO	3.1	2.705 to 3.495	<0.0001
LEO vs. Gentamicin	-1.8	-2.195 to -1.405	<0.0001
REO vs. NEO	1.6	1.205 to 1.995	<0.0001
REO vs. Gentamicin	-3.3	-3.695 to -2.905	<0.0001
NEO vs. Gentamicin	-4.9	-5.295 to -4.505	<0.0001
Pseudomonas aeruginosa			
LEO vs. REO	4.2	3.805 to 4.595	<0.0001
LEO vs. NEO	5.4	5.005 to 5.795	<0.0001
LEO vs. Gentamicin	2.5	2.105 to 2.895	<0.0001
REO vs. NEO	1.2	0.8046 to 1.595	<0.0001
REO vs. Gentamicin	-1.7	-2.095 to -1.305	<0.0001
NEO vs. Gentamicin	-2.9	-3.295 to -2.505	<0.0001
Staphylococcus aureus			
LEO vs. REO	4.1	3.705 to 4.495	<0.0001
LEO vs. NEO	4.3	3.905 to 4.695	<0.0001
LEO vs. Gentamicin	-2.1	-2.495 to -1.705	<0.0001
REO vs. NEO	0.2	-0.1954 to 0.5954	0.5485
REO vs. Gentamicin	-6.2	-6.595 to -5.805	<0.0001
NEO vs. Gentamicin	-6.4	-6.795 to -6.005	<0.0001
Salmonella enterica			
LEO vs. REO	1.5	1.105 to 1.895	<0.0001
LEO vs. NEO	2.1	1.705 to 2.495	<0.0001
LEO vs. Gentamicin	-1.5	-1.895 to -1.105	<0.0001
REO vs. NEO	0.6	0.2046 to 0.9954	0.0008
REO vs. Gentamicin	-3	-3.395 to -2.605	<0.0001
NEO vs. Gentamicin	-3.6	-3.995 to -3.205	<0.0001

Supplementary Table S7. Statistical comparison of the antibacterial activities in terms of minimum inhibitory concentration among different essential oils and gentamicin

Tukey's multiple comparisons test	Mean Diff.	95.00% CI of diff.	Adjusted P Value
Escherichia coli			
LEO vs. REO	-0.37	-0.4026 to -0.3374	<0.0001
LEO vs. NEO	-0.37	-0.4026 to -0.3374	<0.0001
LEO vs. Gentamicin	0.6	0.5674 to 0.6326	<0.0001
REO vs. NEO	0	-0.03258 to 0.03258	>0.9999
REO vs. Gentamicin	0.97	0.9374 to 1.003	<0.0001
NEO vs. Gentamicin	0.97	0.9374 to 1.003	<0.0001
Pseudomonas aeruginosa			
LEO vs. REO	0	-0.03258 to 0.03258	>0.9999
LEO vs. NEO	-0.12	-0.1526 to -0.08742	<0.0001
LEO vs. Gentamicin	0.6	0.5674 to 0.6326	<0.0001
REO vs. NEO	-0.12	-0.1526 to -0.08742	<0.0001
REO vs. Gentamicin	0.6	0.5674 to 0.6326	<0.0001
NEO vs. Gentamicin	0.72	0.6874 to 0.7526	<0.0001
Staphylococcus aureus			
LEO vs. REO	-0.25	-0.2826 to -0.2174	<0.0001
LEO vs. NEO	-0.5	-0.5326 to -0.4674	<0.0001
LEO vs. Gentamicin	0.44	0.4074 to 0.4726	<0.0001
REO vs. NEO	-0.25	-0.2826 to -0.2174	<0.0001
REO vs. Gentamicin	0.69	0.6574 to 0.7226	<0.0001
NEO vs. Gentamicin	0.94	0.9074 to 0.9726	<0.0001
Salmonella enterica			
LEO vs. REO	-0.62	-0.6526 to -0.5874	<0.0001
LEO vs. NEO	-0.62	-0.6526 to -0.5874	<0.0001
LEO vs. Gentamicin	0.6	0.5674 to 0.6326	<0.0001
REO vs. NEO	0	-0.03258 to 0.03258	>0.9999
REO vs. Gentamicin	1.22	1.187 to 1.253	<0.0001
NEO vs. Gentamicin	1.22	1.187 to 1.253	<0.0001
